# Scaling–up public sector childhood diarrhea management program: Lessons from Indian states of Gujarat, Uttar Pradesh and Bihar

**DOI:** 10.7189/jogh.05.020414

**Published:** 2015-12

**Authors:** Sanjeev Kumar, Rajashree Roy, Sucharita Dutta

**Affiliations:** Micronutrient Initiative, New Delhi, India

## Abstract

**Background:**

Diarrhea remains a leading cause of death among children under five in India. Public health sector is an important source for diarrhea treatment with oral rehydration salts (ORS) and zinc. In 2010, Micronutrient Initiative started a project to improve service delivery for childhood diarrhea management through public health sector in Gujarat, Uttar Pradesh (UP) and Bihar. This paper aims to highlight feasible strategies, experiences and lessons learned from scaling–up zinc and ORS for childhood diarrhea management in the public sector in three Indian states.

**Methods:**

The project was implemented in six districts of Gujarat, 12 districts of UP and 15 districts of Bihar, which includes 10.5 million children. Program strategies included capacity building of health care providers, expanding service delivery through community health workers (CHWs), providing supportive supervision to CHWs, ensuring supplies and conducting monitoring and evaluation. The lessons described in this paper are based on program data, government documents and studies that were used to generate evidence and inform program scale–up.

**Results:**

140 000 health personnel, including CHWs, were trained in childhood diarrhea management. During three years, CHWs had sustained knowledge and have treated and reported more than three million children aged 2–59 months having diarrhea, of which 84% were treated with both zinc and ORS. The successful strategies were scaled–up.

**Conclusion:**

It is feasible and viable to introduce and scale–up zinc and ORS for childhood diarrhea treatment through public sector. Community–based service delivery, timely and adequate supplies, trained staff and pro–active engagement with government were essential for program success.

Diarrhea remains one of the leading causes of death among children under 5 in the world, causing 9.2% of total under 5 child deaths [1]. In India childhood diarrhea contributes to nearly 12.6% of all deaths in children below five years [2], which is nearly 2 million child deaths annually. Despite a decline in diarrheal deaths [1], the high mortality and disease burden due to diarrhea underscores the need for urgent attention.

It has been found that zinc when given for the treatment of diarrhea reduces diarrhea mortality by 23%, decreases the severity of the initial episode, and may prevent future diarrheal episodes in the 2–3 months following supplementation [3].

ORS is a simple, proven treatment that can be used to prevent and manage dehydration and decrease in diarrhea mortality. In a meta–anlaysis of ORS intervention studies, 69% pooled relative reduction has been noticed in diarrhea mortality in communities in which ORS was promoted compared with comparison areas. Use of ORS also results in 29–89% relative decreases in referrals to health centres in the intervention areas. [4].

To manage childhood diarrhea and reduce diarrhea–related mortality and morbidity, WHO and UNICEF have recommended the use of both ORS and zinc for diarrhea treatment [5], which has also been adopted by the Government of India. The duration of treatment with zinc for all children in the age group 2–59 months is 14 days and the recommended dosage is 10 mg of zinc per day for children 2–6 months of age and 20 mg per day for children 6–59 months of age, as per these guidelines. Despite this renewed focus, there has been slow progress in operationalizing these guidelines and diarrhea prevalence remains high at 11.7% in India [6]. Zinc is still not being used at scale in combination with ORS under government programs resulting in low coverage of zinc [7].

Micronutrient Initiative (MI) has been a pioneer organization in demonstrating childhood diarrhea management with zinc as an adjuvant to ORS among large populations, and understanding its feasibility for scale–up through the public health sector (including government hospital, primary health center–PHC, sub center, auxiliary nurse midwife–ANM, accredited social health activist–ASHA) and anganwadi workers–AWW). MI has implemented childhood diarrhea management projects in the three Indian states of Gujarat, Uttar Pradesh (UP) and Bihar.

The programs focused on:

• Creating a positive enabling environment, building commitment and sustainability for diarrhea management in the implementation states, and

• Improving availability of supplies at the point–of–care for treating childhood diarrhea

The lessons from these projects along with earlier demonstration projects helped to facilitate the scale–up and mainstreaming of therapeutic zinc supplementation and oral rehydration for treatment of childhood diarrhea through public sector channels. In all three states, the care seeking for childhood diarrhea in private sector is high (**Table 1**). However, scope of this paper is limited to the experiences of implementing childhood diarrhea management program through public sector.

**Table 1:** Situation of childhood diarrhea care–seeking and treatment prior to program implementation in the three states*

	Gujarat (%)	Uttar Pradesh (%)	Bihar (%)
Children under five with diarrhea in the last two weeks (DLHS–3, 2007–08) [6]	11.8	16.2	12.1
Care–seeking for diarrhea from any source (DLHS–3, 2007–08) [6]	65.6	73.8	73.6
Care–seeking for diarrhea in public sector (Public sector includes Government hospital or dispensary, urban health centre/ urban health post/ urban family welfare centre, community health centre or rural hospital, primary health centre, sub–centre, ICDS and Government AYUSH hospital/clinic) (DLHS–3, 2007–08) [6]	43.7	10.4	6.1
Care–seeking in private sector (Private sector includes non–governmental hospital/ trust hospital or clinic, private hospital/clinic and private AYUSH hospital /clinic) [6]	56.0	82.7	56.6
Children suffering with diarrhea treated with ORS (DLHS–3, 2007–08) [8]	36.7	17.4	22
Children suffering with diarrhea not receiving any treatment (DLHS–3, 2007–08) [6]	34.4	26.2	26.4
Use of zinc for diarrhea treatment (among the children having diarrhea in last two weeks) (NFHS–3, 2005–06) [7]	0.0	0.5	0.0

DLHS 3 – District Level Household Survey–3 (DLHS-3), ICDS – Integrated Child Development Services, AYUSH – Department of Ayurveda, Yoga and Naturopathy, Unani, Siddha and Homoeopathy, NFHS 3 – National Family Health Survey 3

*Since program evaluation data are not being used in this paper, data from NFHS 3 (2005–2006) and DLHS 3 (2007–2008) have been used as these are the most recent data available prior to initiation of the projects. For indicator on use of zinc for diarrhea treatment, NFHS 3 data has been used as this indicator is not there in DLHS 3.

Our study aims to highlight: 1) feasible program strategies which could be used to strengthen and scale–up childhood diarrhea management program, and 2) key lessons learned in scaling–up of zinc and ORS treatment for childhood diarrhea through the public sector.

## PROGRAM

The methodology adopted includes use of program monitoring data, findings of research studies, government documents and data from government surveys to provide evidences for key achievement and draw lessons and conclusions. The main sources of data used in the paper include program reporting and monitoring through government system, periodic supply audit studies and other research studies carried out through external research organizations under this program such as rapid assessment of treated child diarrhea cases, caregivers practice and acceptability of frontline workers as credible providers and process evaluation of supportive supervision under childhood diarrhea management program.

Key strategies and challenges faced under the program have been described to give better understanding of implementing the program at scale.

### Program area and demographic information

**Figure 1:**
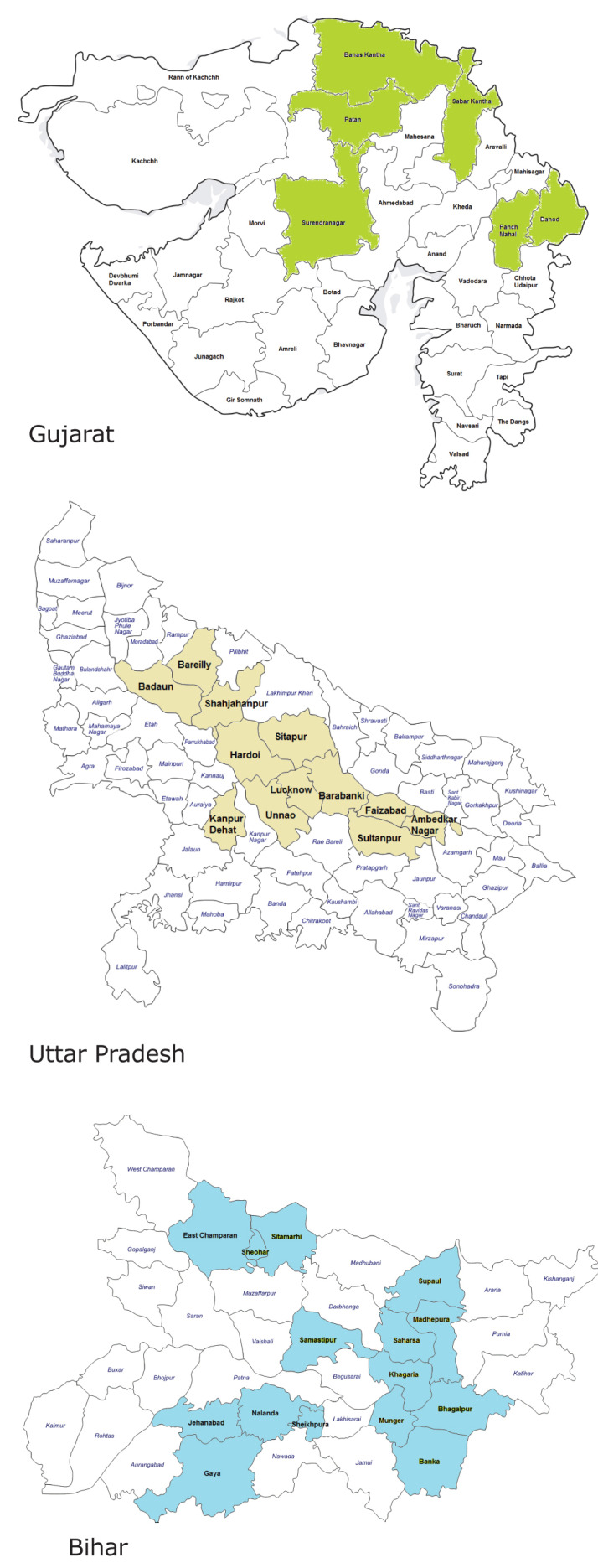
Map of intervention districts.

The project was implemented in UP and Gujarat between 2010 and 2014 and in Bihar between 2010 and 2015. In Gujarat and Bihar, the projects were initially implemented in 6 and 15 demonstration districts, respectively and subsequently scaled–up across the entire state by the respective state governments. In UP, the program was implemented in 12 districts under the Diarrhea Alleviation through Zinc and ORS Therapy (DAZT) project (**Figure 1**). The states and districts were selected based on high prevalence of childhood diarrhea and feasibility of program implementation. **Table 2** further describes the intervention areas. The largest program was in Bihar with 15 demonstration districts and 23 scale–up districts, which reached an estimated population of 13 211 546 children aged 2–59 months.

**Table 2:** Project area description

Program aspect	Gujarat	Uttar Pradesh	Bihar
Donor	USF–BMGF/Teck	USF–BMGF	CIFF
Project name	Diarrhea Alleviation through Zinc and ORS Therapy (DAZT)	Diarrhea Alleviation through Zinc and ORS Therapy (DAZT)	Reducing Deaths from Diarrhea in the Indian State of Bihar
Year of initiation	2010	2010	2010
Year of completion	2014	2014	2015
Geographic coverage	6 demonstration districts 27 scale–up districts	12 demonstration districts	15 demonstration districts, 23 scale–up districts
Target population in the age group of 2–59 months (estimated based on Census of India 2011) [9]	5 370 014	4 338 314	13 211 546

USF – US Fund for UNICEF, BMGF – Bill and Melinda Gates Foundation, CIFF – Children’s Investment Fund Foundation, DAZT – Diarrhea Alleviation through Zinc and ORS Treatment

**DAZT districts of Gujarat:** In Gujarat, the demonstration project was implemented in six out of 26 districts: 1. Sabarkantha, 2. Patan, 3. Banaskantha, 4. Surendranagar, 5. Panchmahal, 6. Dahod.

**DAZT districts of Uttar Pradesh:** In Uttar Pradesh, the demonstration project was implemented in 12 out of 75 districts: 1. Ambedkar Nagar, 2. Kanpur Dehat, 3. Barabanki, 4. Lucknow, 5. Bareilly, 6. Shahjahanpur, 7. Budaun, 8. Sitapur, 9. Faizabad, 10. Sultanpur, 11. Unnao, 12. Hardoi.

**DAZT districts of Bihar:** In Bihar the demonstration project was implemented in 15 out of 38 districts: 1. Banka, 2. Bhagalpur, 3. Samastipur, 4. Sitamarhi, 5. Sheohar, 6. Munger, 7. Madhepura, 8. Sharsha, 9. Khagaria, 10. Supaul, 11. Nalanda, 12. Sheikhpura, 13. East Champaran, 14. Gaya, 15. Jehanabad.

**Table 1** shows the situation of childhood diarrhea care seeking and treatment prior to the implementation of the DAZT programs across the three states. Care–seeking for diarrhea was at 65.6% in Gujarat, 73.8% in UP and 73.6% in Bihar [6]. ORS treatment rates were low at 17.4% in UP, 36.7% in Gujarat and 22% in Bihar [6]; whereas treatment with zinc was negligible in all three states [7].

As can be seen, the demonstration projects were initiated by MI in the three states against this backdrop of high prevalence of child diarrhea, low care–seeking and treatment coverage. In addition, across all three states, ORS and zinc distribution for childhood diarrhea treatment was hampered by budgeting and procurement issues, and there was a lack of systematic health worker training to effectively manage childhood diarrhea cases based on revised national guidelines.

### Key program strategies

The main strategy was to strengthen public sector capacity to manage childhood diarrhea using zinc and ORS at both facility and community levels. Key program components included:

• Building capacity of facility level health providers, and frontline health workers including integrated child development services (ICDS) workers,

• Extending service delivery to communities through frontline workers and at health facilities,

• Ensuring supplies of ORS and zinc at all levels,

• Providing supportive supervision to community health workers,

• Conducting monitoring and evaluation activities.

### Capacity building of health providers

One of the objectives of the program was to ensure enhanced knowledge of service providers on zinc and ORS. This included enabling providers to prescribe zinc and ORS for treating childhood diarrhea and to undertake necessary counseling of caregivers for using these products. A formative research study was conducted in five demonstration districts of Bihar as a part of the program to better understand issues pertaining to knowledge, attitude and practices (KAP) prevalent in the community and among service providers regarding childhood diarrhea management [8]. The Formative study was largely based on qualitative research methods including in–depth interviews with 22 mothers of children under five years, 18 caregivers (grandmothers and adolescent sisters), 21 influencers (experienced mothers and respected community members), 32 block–level health officials (Lady Health Visitors, Block Health Educators, and Medical Officer In–charges), 20 CHWs, 8 doctors and 26 medical store attendants, along with focus group discussions (FGDs) with 20 mothers of children under five years. The study findings reflect knowledge gaps among mothers and caregivers on causes and prevention of diarrhea. The caregivers and service providers found to have poor knowledge on use of zinc for child diarrhea treatment. The findings of this study in Bihar were also utilized in UP and Gujarat. One key gap identified in this qualitative research included knowledge of zinc use for the treatment of childhood diarrhea, and this finding informed the training curriculum for health providers. Specifically, the training curriculum and inter–personal communication (IPC) tools for increasing awareness among caregivers during interaction in one to one and smaller groups were designed for all three states and focused on zinc and ORS and improved case management of dehydration and other related complications. The training modules and methods were further developed for varying capacities of service providers at different levels, and included a training video CD in vernacular and pictorial job aids for conducting the trainings.

An ‘NGO model’ was utilized for the trainings such that professional trainers from select NGOs were hired to train health providers at district and sub–district levels. This was done since medical officers working in the public sector may not have the time or inclination to conduct a large number of trainings in addition to their routine work, and could not necessarily be expected to carry out trainings on such a large scale. However, medical officers were engaged as an expert resource during training sessions. As a result, 144 246 health providers were trained in the intervention districts of UP, Bihar and Gujarat. These providers included medical officers, auxiliary nurse midwives (ANMs), child development program officers (CDPOs), supervisors and anganwadi workers (AWWs) from ICDS and accredited social health activists (ASHAs). All trainings were completed during 2011–2012 except in four UP districts. **Table 3** presents information on the number and types of participants trained in project districts of the three states. Pre and post–tests for participants and monitoring of training sessions were done to ensure quality of the training program. Separate trainings were also conducted for assistant research officers (AROs) and data entry operators (DEOs) in order to improve their understanding on reporting childhood diarrhea cases.

**Table 3:** Details of trained personnel in 2011–2012

Category of personnel	Gujarat (6 demonstration districts)	Uttar Pradesh (12 demonstration districts)	Bihar (15 demonstration districts)
District–level officials, medical officers and CDPOs	673	1736	1757
Block–level health supervisors, ANMs and ICDS supervisors	3547	7079	6864
ASHAs and AWWs	20 198	57 632	44 760
Total	24 418	66 447	53 381

CDPO – Child Development Project Officer, ANM – Auxiliary Nurse Midwife, ASHA – Accredited Social Health Activist, AWW – Anganwadi Worker, ICDS – Integrated Child Development Services

### Extending service delivery to communities

Prior to this program, childhood diarrhea management was predominantly carried out at the facility–level and CHWs were largely restricted to conducting community mobilization work.

One of the major objectives of the program was to enhance the use of zinc and ORS in the public sector for childhood diarrhea treatment. To achieve this, community level health workers including Accredited Social Health Activists (ASHAs) that work for the health department, and Anganwadi Workers (AWWs) that work for the department of woman and child development were involved in the program as service providers to increase public sector care seeking practices. During the program, ASHAs and AWWs were trained and supplied with zinc and ORS to manage diarrhea cases in communities, in addition to facility–based programs. The roles of CHWs included managing simple cases of diarrhea with ORS and zinc, identifying cases of moderate and severe dehydration for referral, and counseling of caregivers on appropriate diarrhea treatment and feeding practices during illness episodes. Thus, in the three states, service was delivered through a network of 66 418 ASHAs, 75 685 AWWs, 9516 health sub centers (HSCs), 918 primary health centers/community health centers (PHCs/CHCs) and 44 district–level hospitals in 33 demonstration districts.

### Ensuring available supplies of ORS and zinc

Ensuring adequate and timely supplies of zinc and ORS was one of the major project strategies, which, along with supply chain management, are essential for ensuring continuous availability of zinc and ORS across all levels of the public health system.

In the beginning, MI introduced initial supplies in the form of combi–packs of zinc and ORS in 10 of the 15 demonstration districts of Bihar, 4 of the 6 districts of Gujarat and 8 of the 12 districts of UP. A total of 3 157 750 combi–packs were distributed. These combi–packs contained 14 tablets of taste–masked dispersible zinc (20 mg each), 2 packets of ORS, one measuring cup and an instruction leaflet. An additional 600 000 zinc tablets were distributed in the remaining 2 districts of Gujarat to be co–packaged with ORS supplies from the Gujarat government. Subsequently, in all the three states, MI facilitated incorporation of budgets under the National Health Mission (NHM) Project Implementation Plan (PIP) for procurement of ORS and zinc. After the first year, the government, in all three states, procured supplies. In Bihar, the state government procured and distributed zinc syrup for the treatment of childhood diarrhea. MI assisted the states to improve forecasting of zinc and ORS demand in order to strengthen procurement based on the child population estimates, diarrhea incidence, and care–seeking rates. It was ensured that states have rate contracts for zinc and ORS in place.

### Providing supportive supervision to CHWs

CHWs require continuous support and guidance to sustain their knowledge levels in order to effectively manage diarrhea cases. To this end, a mechanism of supportive supervision was envisaged through a network of supportive supervisors at the sub–district level. A ‘partnership model’ was followed in UP and Gujarat, where supportive supervision was implemented in partnership with NGOs having a state–level presence, whereas in Bihar this support was provided using the existing cadre of government functionaries at PHCs called Block Community Mobilizers (BCMs) . Supportive supervisors visited CHWs and provided one–on–one support to them and interacted with caregivers to understand their level of adherence to zinc treatment. In addition, they also addressed gaps in CHW capacities during group meetings. Supportive supervisors were provided with a checklist to be completed during supportive supervision visits in order to identify existing knowledge gaps. This checklist captured data on CHW knowledge, skills and availability of supplies.

During each visit, a supportive supervisor was expected to meet at least three field workers and one or two caregivers who had visited CHWs with sick children. In the three states the number of ANM, ASHA, AWWs and caregivers reached through supportive supervision visits is shown in **Table 4**.

**Table 4:** Total ANM, ASHA, AWW and caregivers visited during supportive supervision visits

ANM, ASHA, AWW and caregivers	Gujarat (May 2013–September 2014)	Uttar Pradesh (May 2013–September 2014)	Bihar (December 2011–September 2014)
ANM	3648	2476	6252
ASHA	5500	8578	6237
AWW	5432	7631	6070
Caregivers	12 579	9142	21 984

ANM Auxiliary Nurse Midwife, ASHA – Accredited Social Health Activist, AWW – Anganwadi Worker

The sites visited for supportive supervision were planned each month and priority was accorded to poor performing frontline workers. The findings from supportive supervision visits were used to give one–to–one feedback as well as group feedback during monthly meetings with the frontline workers. Data was analyzed and findings were shared at national, state and district levels to understand program issues and to suggest mid–course corrective actions.

### Conducting monitoring and evaluation activities

A Health Management Information System (HMIS) exists in the public health system for all three states in order to report on public health service delivery. However, HMIS captures data reported by ANMs at health sub–centre and above facilities but do not have a formal reporting tools for CHWs who were also involved in service provision under the project. Hence simple pictorial formats were developed to facilitate childhood diarrhea reporting by semi–literate CHWs. In addition, HMIS did not include indicators to track progress of key program aspects, such as dehydration levels and the number of diarrhea cases treated with both zinc and ORS. Therefore, a project reporting mechanism was developed to effectively track performance from all service delivery points, including CHWs using pictorial formats.

Trainings were provided to health care providers at all service delivery levels, including the CHWs who received close support through the supportive supervision mechanism previously described. A large number of cases were reported through this system from the beginning of the project in all three states. The Government of Bihar has involved CHWs for service delivery in the scale–up districts and has also introduced simple pictorial reporting tools based on the utility of this program reporting mechanism.

In Bihar, the government has added the following new HMIS indicators in that state:

• Number of cases of childhood diarrhea with no dehydration,

• Number of cases of childhood diarrhea with some dehydration,

• Number of cases of childhood diarrhea with severe dehydration,

• Number of cases of childhood diarrhea treated with both zinc and ORS,

• Number of cases of childhood diarrhea referred,

• Number of ASHAs reported ORS stock–out (lack of ORS availability),

• Number of ASHAs reported zinc stock–out (lack of zinc availability).

Currently the program is attempting to streamline and sustain the government HMIS reporting. In addition to reporting, data collected under supportive supervision and research studies also contributed to overall program monitoring in order to understand its progress and to take corrective actions as required. Program evaluation studies planned under this program were conducted by The Johns Hopkins School of Public Health (JHSPH) and results are reported elsewhere [10].

## KEY ACHIEVEMENTS AND LESSONS LEARNED

This section describes key achievements and lessons learned from the programs in three states.

### Ensuring regular availability of zinc and ORS supplies is critical for program performance and needs sustained efforts

**Regular supplies increase diarrhea cases appropriately treated.** Regular availability of zinc and ORS in the public sector is critical for the treatment of diarrhea cases with both zinc and ORS. Program data report that 593 030 childhood diarrhea cases received treatment in 6 demonstration districts of Gujarat between November 2011 and September 2014. More than 99% of these cases were treated with both zinc and ORS. In 12 demonstration districts of UP, 907 295 diarrhea cases reportedly received treatment between December 2011 and September 2014, and 86% of these cases were treated with both zinc and ORS. In Bihar, 1 796 563 cases were reported to have received treatment in 15 demonstration districts between August 2011 and September 2014, and 77% were treated with both zinc and ORS. Importantly, public sector service providers in intervention districts reported only those cases of childhood diarrhea that were provided with any treatment and cases not given treatment were not reported. In Gujarat, the higher percentage of childhood diarrhea cases treated with both zinc and ORS was largely due to the uninterrupted availability of zinc and ORS in that state compared to UP and Bihar that had supply interruptions (**Table 5**). A baseline study on caregiver’ knowledge, attitude and practice on the use of zinc and ORS for childhood diarrhea in Bihar also reflects that availability of zinc and ORS with service providers is one of the reasons that prevents treatment seeking by caregivers for childhood diarrhea from such service providers [11].

**Table 5:** Stock–outs and diarrhea cases treated with zinc and ORS in the public sector

District	ASHAs having stock–out* of zinc and ORS (%)	Total number of child diarrhea cases brought to public sector for care and received any treatment	Total number of child diarrhea cases who received both zinc and ORS†	Child diarrhea cases seen in the public sector and treated with both zinc and ORS (%)
Gujarat‡	3	593 030	590 552	99
Uttar Pradesh§	48	907 295	778 970	86
Bihar¶	28#	1 796 563	1 374 869	77

ORS – oral rehydration salts

*Stock out – lack of zinc/ORS availability.

†Childhood diarrhea cases treated with zinc and ORS are based on program reports. Gujarat cases refer to November 2011–September 2014; Bihar refers to August 2011–September 2014; UP refers to December 2011–September 2014.

‡Gujarat stock–out from May 2013–September 2014 is based on supportive supervision data.

§UP stock–out from May 2013–September 2014 is based on supportive supervision data.

¶Bihar stock–out is based on supply audits conducted in January 2012, June 2012, October 2012, March 2013 and February 2014.

#After March 2014 there was zinc stock out with most of the service providers.

These findings suggest that availability of regular supplies of zinc and ORS helps in treating a higher percentage of diarrhea cases with both zinc and ORS.

**Ensuring regular supplies needs sustained efforts.** The maintenance of regular zinc and ORS availability at different service delivery points has proved a challenge in the public sector, and particularly in states with relatively poor governance. In Bihar, supply audit studies were periodically conducted to assess zinc and ORS availability among CHWs. The supply audits indicated that there were no stock–outs in the first six months of supply distribution but thereafter 28% of ASHAs on average experienced stock–outs of both zinc and ORS between June 2012 and February 2014. After April 2014, there was a zinc stock–out at most service delivery points due to expiry of existing zinc syrup and other challenges faced by the state during the process of ensuring new procurement mechanisms. Similarly, in UP, ORS was unavailable at many of the services delivery points at different time periods between November 2013 and October 2014 due to procurement challenges.

MI and other development partners made sustained efforts to restore supplies in both Bihar and UP, such as through advocacy for placement of procurement orders, providing technical support for accurate demand forecasting, advising on product specifications, and providing close support in supply distribution to service delivery points. Gujarat already had an improved supply chain mechanism that resulted in fewer ASHAs experiencing zinc and ORS stock–outs (**Table 5**).

Indeed, ensuring regular supplies is one of the biggest challenges in the public health system not only in India but across the countries for treating childhood diarrhea with both zinc and ORS [12]. The important gaps that need to be addressed to ensure regular zinc and ORS availability include:

• Lack of knowledge among program managers in the public health system for forecasting the required quantity of drugs: In general, public sector supplies are procured without accurate demand calculations and therefore training and technical support for proper forecasting is important for procuring the adequate supply quantities [13].

• Complicated and time–consuming procurement processes: procurement is carried–out by the state government, for which getting the information on quantity required from various levels, placing procurement orders, testing drug quality and appropriate distribution is cumbersome and time–taking. Recognizing this problem, some of the states have engaged a dedicated corporation for their drug procurement and distribution. However, in some states such corporations are either newly established or not fully operational. Therefore technical support provided at different procurement stages could facilitate the process until these procurement corporations become fully functional.

• Lack of proper warehousing facilities at district and sub–district levels: Due to lack of proper storage facilities the districts and blocks (sub–districts) are unable to store supplies required for replenishing unanticipated stock–outs [14].

• Lack of an effective mechanism of tracking availability of supplies with CHWs and timely replenishment of stocks at service delivery points in the case of stock outs: The HMIS and drugs logistic management system (DLMS) does not track CHW supply availability, which is a major impediment for replenishing CHW stocks [15].

### Regular supplies promotes caregiver confidence in CHWs and public health sector

Lack of available zinc and ORS weakens caregiver trust and reliance in the quality of public health care services, which adversely affects care–seeking at the public sector. A recent study [16] on care–seeking for childhood diarrhea and acceptance of CHWs as credible service providers in the demonstration districts in Bihar, found reduced trust among caregivers on the quality of care and services provided by CHWs, as a result of lack of zinc and ORS availability with them. In public health sector, frequent stock–outs also weaken the confidence of CHWs to provide treatment of child diarrhea. Similar findings were also observed in a study on health care reforms involving the introduction of user fees and drug revolving funds and their influence on health workers’ behavior in Nigeria [17].

These results indicate the importance of regular zinc and ORS availability for increased public sector care seeking by improving the credibility of CHWs among caregivers and boosting confidence of the CHWs to act as well–equipped service providers.

### Quality capacity building activities ensures sustained knowledge and skills of the health functionaries

A large number of health providers were trained in the program as previously described. These trainings were conducted with the help of external trainers who were supported technically by government medical officers, which facilitated the completion of trainings within the stipulated short timeline and with quality assurance. Though baseline data on CHW knowledge level is not available, data collected during supportive supervision visits provide information on knowledge levels of CHWs. During supportive supervision visits, information on CHW knowledge and capacity was collected using a checklist by block community mobilizers (BCMs) in Bihar and by NGO partners in Gujarat and UP. In Bihar nearly 100 BCMs placed at block level in 10 districts undertook a maximum of four supportive supervision visits each month. In Gujarat and UP, 20 and 27 supportive supervisors, respectively, undertook field visits for approximately 20 days in each month. During each visit, a supportive supervisor was expected to meet and collect data from at least three field workers (1 ANM, 1 ASHA and 1 AWW) and to meet with one or two caregivers who attended CHW services. These data were analyzed on a monthly basis and generally showed sustained CHW knowledge and skills, which indicates the good quality of training provided under the program (**Table 6**). **Table 6** highlights ASHA awareness level regarding the definition of diarrhea (three or more watery stool within 24 hours) was either sustained or improved. Similarly, there was sustained or increased ASHA awareness about the age–wise correct dosage of zinc across the three states (**Table 7**). An end–line evaluation study of caregivers’ knowledge, attitude and practice on the use of zinc and ORS for childhood diarrhea, carried out in 11 blocks of five districts of Gujarat, where trainings were conducted using similar methods that were adopted under the DAZT program districts, also show a high and sustained knowledge of ASHA on identification of childhood diarrhea and its management protocols [18].

**Table 6:** ASHA knowledge of diarrhea definition

District	May 2013		November 2013		May/June 2014		September 2014	
**Number of ASHAs visited**	**ASHA knows definition of diarrhea (%)**	**Number of ASHAs visited**	**ASHA knows definition of diarrhea (%)**	**Number of ASHAs visited**	**ASHA knows definition of diarrhea (%)**	**Number of ASHAs visited**	**ASHA knows definition of diarrhea (%)**
Gujarat	315	94	208	99	307	95	176	90
Uttar Pradesh	366	51	443	81	552	81	585	94
Bihar*	211	88	196	94	51	90	141	89

ASHA – Accredited Social Health Activist

*In Bihar supportive supervision data shown in May/ June 2014 column refers to data collected in June 2014. The activity was halted for about two to three months (April to mid–June 2014) to make necessary changes like role out of new formats, training of supervisors etc as a result number of ASHAs visited are less. For other states the data pertains to May 2014.

**Table 7:** ASHA knowledge about age–wise zinc doses

District	May 2013		November 2013		May/June 2014		September 2014	
**Number of ASHA visited**	**ASHA knows age wise dose of zinc (%)**	**Number of ASHA visited**	**ASHA knows age wise dose of zinc (%)**	**Number of ASHA visited**	**ASHA knows age wise dose of zinc**	**Number of ASHA visited**	**ASHA knows age wise dose of zinc (%)**
Gujarat	315	87	208	92	307	91	176	96
Uttar Pradesh	366	52	443	69	552	54	585	71
Bihar*	211	86	196	95	51	86	141	83

ASHA – Accredited Social Health Activist

*In Bihar supportive supervision data shown in May/June 2014 column refers to data collected in June 2014. The activity was halted for about two to three months (April to mid–June 2014) to make necessary changes like role out of new formats, training of supervisors etc as a result number of ASHAs visited are less. For the other states the data pertains to May 2014.

### Involvement of CHWs strengthens service delivery and performance reporting

**Figure 2:**
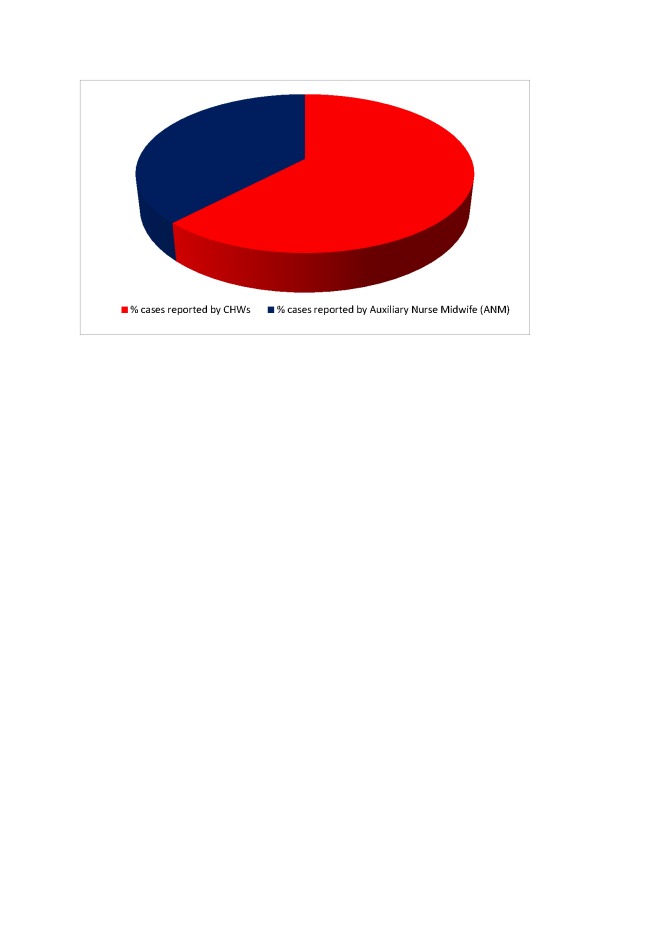
Proportion of cases treated and reported by community health workers (CHW) and auxiliary nurse midwives (ANM) in Bihar, May 2013.

**Enhances percentage of cases treated at community level.** A key program strategy was to involve CHWs in the management of child diarrhea cases and to report program performance. Availability of services increased at the community level by involving CHWs in service delivery. In Bihar, 62% of non–facility cases seen by ANMs and CHWs were treated and reported by CHWs. This indicates better availability of services and easy access by caregivers near their homes. A similar pattern of CHW treatment and reporting was also found in UP and Gujarat (**Figure 2**). Evalutation of DAZT program in Gujarat reflects increased coverage of diarrhoea treatment by CHWs [10].

**Figure 3:**
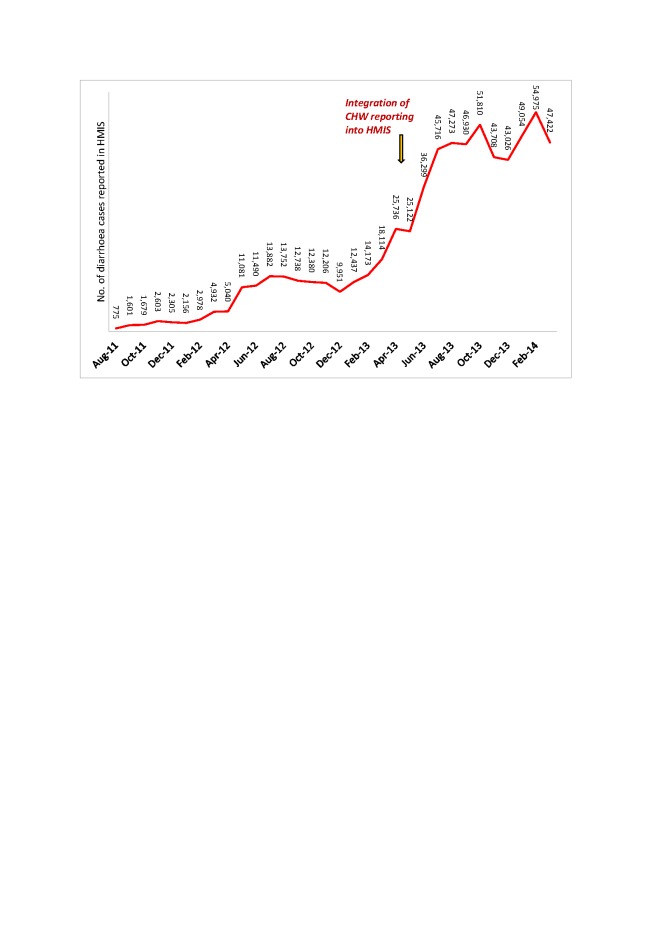
Diarrhea cases reported in 15 demonstration districts of Bihar before and after integrating the community health worker (CHW) reporting system into Health Management Information System (HMIS).

**Improves tracking program performance and increases reporting of child diarrhea cases.** Along with service delivery, CHWs were also involved and trained for performance reporting. The public health system has traditionally focused on facility–based data and reporting. In this program, CHWs were also involved in reporting in order to track the treatment of cases at the community level. As previously described, a simple pictorial format was developed to facilitate CHW reporting and CHW capacities for reporting were reinforced through supportive supervision visits. The number of reported cases increased after involving CHWs in the reporting process. **Figure 3** shows the increased number of childhood diarrhea cases in Bihar both before and after CHW reports were integrated into HMIS in that state.

Reporting by CHWs however requires further strengthening. Reporting completeness exercises conducted in the three states indicated that only 41% of ASHAs in Bihar (May 2013), 57% in UP (July 2013) and 82% in Gujarat (June 2013) were able to report cases using the standard form, and the remaining were not able to submit the report. CHW report submission can be further improved by streamlining the reporting mechanism, ensuring availability of reporting tools and close supervision to enhance their capacity on case reporting. A review of integrated community case managemnt in sub Sahara African countries also highlights that involvemnt of reporting by CHWs can strngthen HIMS, if CHWs are provided simple reporting tools and have a few selective indicators to report [19].

**Enhances caregiver access and reach of public health services for childhood diarrhea management.** Involving CHWs in service provision for childhood diarrhea management helps increase caregivers access to services and the reach of the public health system. A rapid assessment study conducted by MI in Gujarat and UP suggested that caregivers prefer receiving free–of–cost services closer to home from CHWs [20]. Making the community aware about the availability of zinc and ORS with CHWs, coupled with strong CHW skills and training in the management of child diarrhea, can further enhance the acceptability of CHWs by the community. This in turn will help increase zinc and ORS coverage through the public sector.

Probably a component of behaviour change communication as a part of program strategies would have contributed to increase in caregivers’ awareness thereby resulting in greater demand for zinc and ORS for management of childhood diarrhea.

### Continuous engagement with government and sharing early evidences of program performance facilitated government decision–making for scale–up

In order to ensure government ownership from the program outset, attempts were made to continously engage the government at all levels. At the start of the program, Memoranda of Understanding (MOUs) were signed with the state governments of Gujarat and Bihar. The program was also reviewed regularly at the block, district and state levels, which helped strengthen service delivery. In addition, information on program performance was also shared regularly with districts and state level authorities. This continuous engagement with the government and regular sharing of progress achieved has been important for ensuring government ownership, and has paved the path towards the program scale–up.

For the purpose of scale–up, the program have focused since its outset in generating evidence of program performance, ensuring buy–in from the government on program strategies, and enhancing capacity of government functionaries. Generating early evidence on effectiveness and regularly sharing these updates with government counterparts has facilitated the government’s decisions for scale–up of specific program strategies. Despite the challenges in procurement, affecting the program achievements, in UP and Bihar the following strategies have been scaled–up by all the three states to facilitate program expansion across districts [21]:

• CHW involvement in service delivery is now implemented across the entire states of UP, Bihar and Gujarat

• Estimation of zinc and ORS stocks and supply provision to all service delivery points

• Regular reporting mechanisms of cases through the HMIS in Bihar

• Service provider trainings in childhood diarrhea treatment using ORS and zinc

The state governments have earmarked budgets for the procurement of zinc and ORS across districts in order to scale–up program strategies. Bihar, Gujarat and UP governments have incorporated these program activities in their annual plans and have submitted budget proposals to the Government of India for implementation across all districts. The Government of India has approved these proposed budgets for 2012–2013 and subsequent years [22].

### Regular technical support and evidence–based policy advocacy leads to policy changes and release of implementation guidelines

The advocacy efforts undertaken and technical support provided by MI and other development partners at national levels have resulted in the development of operational guidelines by the Ministry of Health and Family Welfare, Government of India [23]. These guidelines include strategies used in the demonstration districts that will help facilitate roll–out of program strategies across all states of India. The advocacy efforts of MI and other partners have also facilitated the following policy changes that will enhance the availability of zinc supply in India:

• Zinc specifications included in the Indian pharmacopeia: Due to this change more Indian pharmaceutical companies can start production of therapeutic zinc [24]

• Zinc included in the essential drug list: The Government of India has included zinc in the list of essential medicines [25]. This will help to ensure zinc availability at service delivery points in the public health sector

• Indicators on zinc usage included in large–scale national surveys (eg, Annual Health Survey of India) [26] and District Level Health Survey–4 [27]): This will help the government track the use of zinc for child diarrhea management at the national and sub–national levels

### Limitations

Information on certain aspects of program has been collected only during the implementation of the projects across the three states and detailed information on these aspects, like stock out of zinc and ORS, reporting of the cases treated with zinc and ORS, cases of childhood diarrhea treated by CHWs, knowledge level of CHWs on management of childhood diarrhea is not available prior to the initiation of the project implementation. Since data collected during the evaluation studies is being published as a separate paper on evaluation findings in this same issue of the journal, hence evaluation data has not been used in this paper. This paper is largely based on program monitoring and data collected through government functionaries which may have some limitations.

## DISCUSSION AND CONCLUSIONS

This program shows that management of childhood diarrhea using zinc as an adjuvant to ORS when implemented through CHWs in addition to improved services provided through public health facilities, leads to increased service accessibility by the community and increased credibility of the public health system resulting in increased coverage [10].

Prior to the initiation of these projects, the use of zinc and ORS for management of childhood diarrhea was not a focused intervention under the government system in the program states. The exposure of government functionaries on use of zinc and ORS for management of childhood diarrhea was therefore limited and the overall use of zinc was low in all the three states. Some of the critical strategies which were implemented across the states like facilitating procurement of supplies by government, capacity building of service providers and managers across cadres, introduction of simple reporting tools for CHWs, program monitoring and review helped in improving knowledge of service providers and increasing uptake of zinc and ORS.

The professional trainings imparted through the program improve CHW capacities and enhanced their skills and knowledge thereby increasing their acceptance in the community as health service providers. However, such outcomes are possible only if there is government ownership of the program, quality training of health service providers, timely and adequate procurement of quality products, timely distribution, an efficient tracking mechanism to identify stock–outs for replenishment, routine monitoring and critical reviews at all levels regarding quality of training provided to service providers.

The strategies adopted under the program have resulted in increased utilization of public health services and use of zinc and ORS for childhood diarrhea treatment. These program outcomes could have further increased if these were supported by activities to generate community awareness on use of zinc and ORS for childhood diarrhea and its availability at public health providers. However, the program did not have any Behaviour Change Communication (BCC) component, other than interpersonal communication, as a part of program strategies. Inclusion of behaviour change communication strategies would have contributed to increase in caregivers’ awareness thereby resulting in greater demand for zinc and ORS for management of childhood diarrhea. This has also been substantiated by an evaluation study on caregiver awareness generation activities carried out in 11 blocks of five districts of Gujarat which reflects increased coverage of zinc and ORS for childhood diarrhea at public sector as a result of caregiver awareness generation activities [18].

This program experiences reflect that it is feasible and viable to introduce and scale–up therapeutic zinc supplementation as an adjuvant to ORS in the management of childhood diarrhea through the public sector. Some of the components which are likely to be sustained beyond the program period under the donor funded childhood dairhoea management program in the three states include knowledge level of service providers, engagement of community health workers for service delivery and reporting through HMIS. It is because some of these components have got included in the government program implementation plan and Government of India has developed and rolled out operational guidelines for childhood diarrhea management and has launched the programs such as India Action Plan for Pneumonia and Diarrhea (IAPPD) and Intensified Diarrhea Control Fortnight (IDCF) which has given significant impetus and brought renewed focus on Childhood diarrhea management.

Demonstration projects like the one reported in this paper, play a critical role in generating early evidence to influence government decisions for scale–up of programs and accelerate necessary policy changes.
